# Chewing Function with Efficiency Tests in Subjects Wearing Clear Aligners

**DOI:** 10.3390/dj12030057

**Published:** 2024-03-01

**Authors:** Luca Levrini, Alessandro Deppieri, Andrea Carganico, Giorgia Rodigari, Stefano Saran, Piero Antonio Zecca, Marco Cicciù, Salvatore Bocchieri

**Affiliations:** 1 Department of Human Sciences, Innovation and Territory, Post Graduate School in Orthodontics, University of Insubria, 21100 Varese, Italy; luca.levrini@uninsubria.it; 2 Department of Medicine and Surgery, Post Graduate School in Orthodontics, University of Insubria, 21100 Varese, Italy; acarganico@studenti.uninsubria.it (A.C.); grodigari@studenti.uninsubria.it (G.R.); ssaran@studenti.uninsubria.it (S.S.); 3 Department of Medicine and Technological Innovation, University of Insubria, 21100 Varese, Italy; pieroantonio.zecca@uninsubria.it; 4 Department of General Surgery and Surgical-Medicial Specialties, University of Catania, 95124 Catania, Italy; mcicciu@unime.it; 5 Department of Biomedical and Dental Science, Morphological and Functional Images, University of Messina, 98122 Messina, Italy; sbocchieri@studenti.uninsubria.it

**Keywords:** orthodontics, dentistry, mastication, dental occlusion, chewing gum, humans

## Abstract

This study assessed the masticatory function of participants wearing clear aligners in order to determine whether these devices can be worn even when eating and therefore worn to extend treatment time and boost treatment effectiveness. An intercontrol test was conducted on 20 patients who received Invisalign^®^ treatment. Each participant was instructed to chew two pieces of Hue-Check Gum^®^ chewing gum (one pink and the other blue) in 5, 10, and 20 cycles both with and without aligners. After being removed from the oral cavity, the gum was dried and pressed using a 1 × 50 × 50 mm model that was 3D printed with a transparent layer in between. After being scanned on both sides with a flatbed scanner at 600 dpi, the samples were saved as jpg files and subjected to an optoelectronic examination using ViewGum software. To validate the procedure, a control group from a different institution (University of Bern) was used. A statistical analysis of the data was carried out. The Shapiro–Wilk test was used to confirm the normality of the samples. A one-way ANOVA test, a homogeneity of variance test, and a t-test did not reveal statistically significant differences between the two control groups, thus validating the methodology employed. In summary, clear aligners do not radically change the masticatory function while they are worn. As a result, clinicians can exploit the aligners for chewing to obtain a better fit of the plastic material to the dental surface and to attachments. Treatment times for patients could also be shorter.

## 1. Introduction

Orofacial functions include chewing, swallowing, talking, laughing, and smiling. All of the organs and structures involved in chewing, such as the teeth, the periodontal tissue, the maxillary bones, the jaw, the temporomandibular joints, the muscles, the nerves, the tongue, the lips, the cheeks, and the oral mucosa, have to cooperate with each other in order to function properly. The effectiveness (achieving the goal) and efficiency (using less resources, time, and energy) of oral functions might change as a result of imbalances between various components, thus having significant effects on health and quality of life.

The masticatory function aids in the breakdown of food into smaller pieces so that they can be easily swallowed and digested, which is essential for good digestion and nutrition. Enzymes that aid in digestion are released into the saliva as a result of chewing [1]. The masticatory function also helps maintain the health of the jaw and teeth. Chewing regularly preserves bone density and accelerates bone formation by stimulating the jaw muscles and temporo-mandibular joint. By enhancing blood flow and avoiding dental decay and gum disease, chewing also keeps the teeth and supporting tissues healthy [2].

A person’s quality of life may be adversely affected by masticatory function impairments, which can cause problems with chewing, swallowing, and speaking. TMJ (temporomandibular joint) issues, muscle wasting, dental issues, and neurological abnormalities are a few common causes of a decreased masticatory function. Depending on the underlying cause, physical therapy, dental procedures, or surgery can be used to treat a reduced masticatory function [3]. The coordination of the jaw muscles, teeth, and tongue is a crucial component of the masticatory function. Food is ground and mixed with saliva when it is placed between the teeth, which is accomplished by the tongue. The teeth are vital in reducing food into tiny pieces that are simpler to swallow and digest [4]. The sensory system, in addition to the muscles, teeth, and tongue, is very important for mastication. The force and length of the chewing cycle are controlled by the brain, which receives information from the sensory receptors in the mouth concerning the texture, consistency, and flavor of the food. By ensuring that the food is adequately chewed before swallowing, this feedback loop lowers the danger of choking and enhances the digestive process. The masticatory function involves a number of interconnected systems and is crucial to maintain general health and wellbeing [5].

Functionality and esthetics are the two main considerations in orthodontic treatment using clear aligners. The orthodontist must treat functional problems while also focusing on esthetics in order to guarantee patient satisfaction. Patients accept clear aligners more frequently due to their comfort and esthetics, as well as the fact that they are less conspicuous and easier to maintain than fixed braces. The wide availability of clear aligners, which are less noticeable than traditional brackets and in higher demand among adults, is probably responsible for the rise in demand for orthodontic procedures. The greatest advantage of clear aligners relies in the possibility of maintaining better oral hygiene, with better periodontal health during orthodontic treatment. A recent publication observed how it is possible to chew with clear aligners, maintaining an efficient masticatory function [6,7].

Maintaining proper dental hygiene is crucial during orthodontic treatment. Poor oral hygiene can cause enamel demineralization with both fixed orthodontic and clear aligners. In this case, the benefits of orthodontic therapy could be compromised by the demineralization of the enamel surface next to orthodontic appliances, which first appears as white spot lesions. To reduce tooth decay and tooth stains, which could impair the esthetics of the smile, it is essential to prevent, diagnose, and treat these issues [8]. Fixed orthodontic appliances facilitate the deposition of bacterial plaque and restrict the patients’ ability to properly care for their teeth, thus increasing the chance of acquiring white spot lesions. Moreover, oral bacterial counts rise after orthodontic bracket positioning. The more appliances patients have bonded in their mouth, the greater the rise in bacterial counts [9]. For the best results, clear aligners should typically be worn for 22 h per day. Saliva’s inherent cleaning and remineralizing properties are negated by the protective environment a plastic aligner generates. Further plaque creation and entrapment under the appliances are due to the fact that the lips, cheeks, and tongue cannot carry out their normal cleansing functions [10]. However, good daily hygiene and the use of the right cleaning techniques, such as the traditional method of brushing with toothpaste and cleaning the aligners with tablets containing sodium carbonate and sulfate, significantly lower the risk of the development of white spots [11,12].

This work tries to emphasize that the chewing capacity is improved, without compromising the kind of diet, by keeping good dental cleanliness even throughout orthodontic treatment (particularly in patients who use transparent aligners). In reality, there is a decreased chance of developing carious and gingival lesions, which may indicate the loss of dental components and ultimately jeopardize the proper function of chewing.

The aim of this study was to assess the masticatory function of patients who have received clear aligner therapy so they can avoid aligner removal during meals and extend the amount of time they wear the aligners, which could lead to a consequent improvement in the results of the treatment.

## 2. Materials and Methods

This study was carried out at the orthodontic department of the University of Insubria in Varese, in collaboration with the University of Bern. A total of 20 participants were enrolled in the study (7 females and 13 males) with complete dentitions (at least 28 teeth), the maximum DMFT index (decayed, missing, and filled teeth) of 4, and the absence of temporomandibular disorders. 

**Figure 1:**
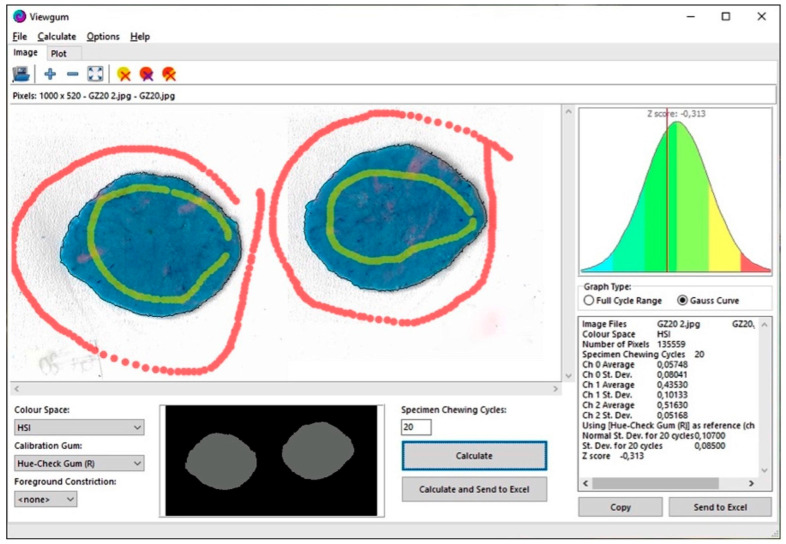
Work screen of Viewgum©.

The technique chosen was explained to participants and entails the use of a blue and a pink chewing gum (Hue-Check Gum^®^, Orophys GmbH, Muri b. Bern, Switzerland). Each subject was positioned upright, and before chewing, the two gums were moistened and placed in the patient’s mouth with the blue side facing the tongue. The patient was then instructed to complete a test of 5, 10, and 20 cycles with and without the clear aligners, with a one-minute break between each test to prevent muscular fatigue. Based on the jaw’s movements, the operator measured the number of masticatory cycles. After removal from the mouth cavity, the chewing gum was dried and pressed into a 1 × 50 × 50 mm 3D-printed mold while being positioned between a transparent sheet. The samples were scanned on both sides using a flatbed scanner (HP LaserJet Managed MFP E72525, Hewlett-Packard, Palo Alto, CA, USA) at 600 dpi. The scansions were then exported as .jpg files and were examined optoelectronically using ViewGum software (Figure 1).

The images were then processed by removing the backdrop surface and a study of the color variation (VOH scan) was carried out. Pictures representing the front side and back side were loaded at the same time. In the HSI color space, hue is a corner; hence, the circular VOH is defined as 1 (the length of the mean vector). The standard deviation (SD) between the two color peaks is shown by ViewGum: SD = sqrt (VOH). VOH has a logarithmic relationship with the quantity of chewing cycles, and it is regarded as a metric of chewing performance. Poor chewing leads to poorly mixed colors and a high VOH, whereas good chewing produces well-mixed colors and a low VOH. Data were then acquired and exported to Excel (Microsoft Corporation, Redmond, Washington, DC, USA). 

Jamovi software was used for the statistical analysis of the results (v. 1.6.14, Jamovi Project, Sydney, Australia). A descriptive analysis was carried out for all the variables. The Shapiro–Wilk test was performed to assess the variables’ normal distribution. The *t*-test for paired samples was used to look for statistically significant differences. The statistical power was set to 90%, and the sample size was estimated based on the work by Buser et al. [1]. The significance level was set to 5%. The results were then compared with a second control group, which was registered in a different institution (University of Bern, Switzerland), to validate the procedure.

## 3. Results

A statistical analysis of our results revealed the regularity of the samples and the fact that Hue-Check Gum^®^ tests produced noticeably different color mixing outcomes based on the quantity of chewing cycles (Table 1 and Table 2).

On the other hand, a statistical analysis of the results showed no statistically significant differences for mixing or for the other parameters examined by the program when comparing the groups with and without aligners with the same number of masticatory cycles (Table 3, Table 4, Table 5, Table 6, Table 7 and Table 8).

The study was validated by comparing our results (i.e., the University of Insubria) with the sample of the University of Bern. The Bern control group served to validate the method. Since the University of Insubria control group was comparable to the Bern group (both recruited students of similar ages), it was decided to retain both. The descriptive statistics are shown in Table 9. (Group 1 stands for the University of Bern’s control sample, Group 2 stands for the University of Insubria’s control sample and Group 3 stands for the University of Insubria’s test sample.)

**Figure 2:**
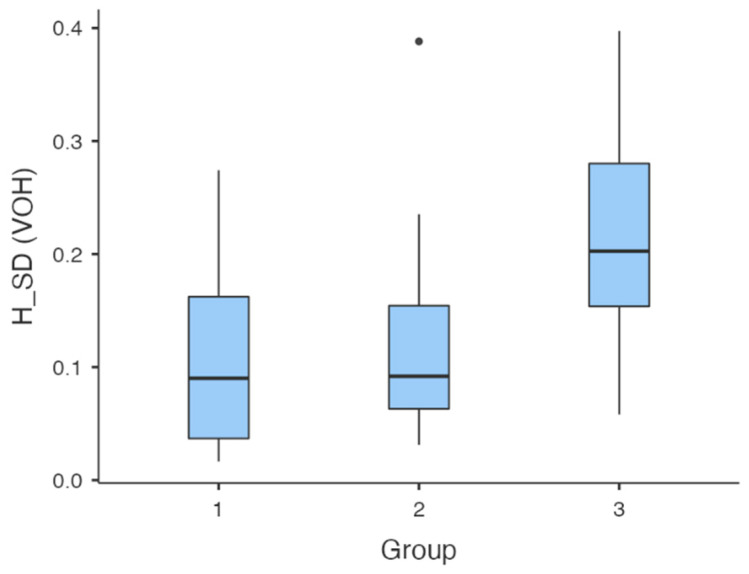
The median and distribution of data in the comparison of VOH between groups 1, 2 and 3 at 20 mastication cycles.

Figure 2 shows the distribution of the VOH value among the groups and highlights that the control groups (1 and 2) overlap, whereas Group 3 is outside the visual range of the control groups.

The results of the ANOVA test and the homogeneity of the variance test are shown in Table 10 and Table 11. Only the VOH parameter, which indicates the difference in chewing ability with and without aligners, showed differences that are statistically significant. 

Table 12 shows the *t*-test between Groups 1 and 2. Because there were no statistically significant differences between the two groups, the procedure is considered to be reproducible and validated, allowing us to compare the two control samples.

## 4. Discussion

The demand for clear aligner treatment has constantly grown during the last decade due to the possibility of treating adults and adolescents with a more esthetic and comfortable alternative. Unlike other orthodontic procedures, clear aligners are removable, simplifying the oral hygiene procedure and resulting in better periodontal health. The manufacturers recommend wearing the aligners from 20 to 22 h every day. To be as effective as possible, orthodontic therapy should be constant and uninterrupted. Ideally, orthodontic pressure should be applied continuously to achieve the best level of effectiveness [13,14]. 

However, in patients receiving orthodontic treatment, a lengthy treatment period without sufficient oral hygiene might lead to the development of white spots. According to Lucchese et al. and Sheridan et al., therapy with fixed appliances and clear aligners can both cause the enamel to deteriorate and lead to white spots. On the other hand, maintaining a proper oral hygiene during treatment when using clear aligners can lower the visibility of white spots and keep the dental enamel healthy [12,15,16]. Studies on clear aligners’ impact on enamel have been conducted in the literature. In terms of time and conditions, wearing the aligners from morning to evening without taking them off during lunch is comparable to the hypothesis of wearing them from evening to morning without taking them off at night [17,18].

The oro-facial system’s function of chewing is exceedingly complex, and a number of elements must work together to break down food into a bolus that is safe to swallow. The number of occluding pairs of teeth may be the most significant predictor of chewing efficiency, although other factors such as maximal force, saliva flow rate, prosthetic reconstruction, force and coordination of the tongue and cheeks, age, and gender are also significant. Chewing function is also correlated with intraoral sensitivity and cognitive state. It is challenging to take all these confounders into consideration [19,20,21]. To assess masticatory performance, a variety of food tests can be used, each with unique physiological properties. Natural food requires a chewing action that occurs naturally, but it may also trigger the instinct to swallow. Additionally, natural food is often unstable when stored for an extended period of time. On the other hand, artificial materials like silicone lead to chewing that is more conscious than natural, but the sample remains stable for a very long time. The use of chewing gum has many benefits, including availability, reproducibility of consistency, affordability, and ease of stocking and long-term storage without affecting the substance’s characteristics [22,23].

The current study showed that there are no appreciable differences in masticatory function between those wearing clear aligners and those not wearing them. Patients were also able to notice an improvement in how well the plastic material fits the dental surface and the attachments, but more thorough research is required to establish which meals are most effective at boosting adhesion. The number of chew cycles used in this investigation was selected to account for all potential levels of color mixing. According to several studies in the literature, the appropriate number of masticatory cycles for bolus mixing tests is on average 20; this threshold can be used to assess chewing performance [24,25,26,27].

It is well acknowledged that two-tone material mixing tests are a reliable method to assess masticatory function [1]. Hue-Check Gum^®^ tests are rapid, and the two-color material mixing procedure is easy to use and does not require specialized equipment or highly expert operators. The examinations are quick and simple to complete, and the subject being tested is not uncomfortable. The sample is fully recovered and, unlike fragmentation tests, the patient does not need to spit out the material repeatedly; there is thus no chance of major sample loss or discomfort from lengthy, possibly uncomfortable examinations.

Due to Hue-Check Gum^®^’s similar characteristics to commercial chewing gum, the subject recognizes it as an inedible product and is not forced to swallow it during the test, as would normally occur when consuming natural foods. As a result, there is no risk of material loss.

The study covers several important facets of clear aligner therapy and how it affects chewing function and oral health. It is evident that it is crucial to make sure that using transparent aligners during orthodontic treatment does not impair one’s ability to chew food. The study shows that, in spite of worries about possible adverse effects on the adherence to clear aligners, the masticatory function basically stays the same during and after treatment. This outcome is crucial because it shows that patients receiving treatment with clear aligners can continue to chew normally, ensuring that oral functions that are critical to overall health and wellbeing are performed correctly.

Additionally, orthodontists and patients can rest easy knowing that chewing function is unaffected by clear aligner treatment. Patients may be sure that their treatment will not make it harder for them to chew or speak, and orthodontists can feel comfortable recommending this course of action without worrying about how it will affect their patients’ ability to chew.

In conclusion, one of the most important factors in modern orthodontics to take into account is the patient’s ability to chew during treatment with clear aligners. Both patients and orthodontists stand to gain much from confirmation that this function mostly stays the same throughout and after treatment, since it guarantees both an improved quality of life and secure and efficient orthodontic care.

However, there are factors that limit this research. These are primarily linked to the number and age of the sample. Conducting subsequent studies with a larger number of patients and a wider age range can certainly make the study more meaningful. Studying finite elements would also be necessary to determine whether the several chewing cycles that transparent aligners endure erode the aligner’s structure or the therapeutic support components (such attachments).

## 5. Conclusions

This study highlighted that clear aligners do not significantly alter the masticatory function while they are worn. As a result, a clinician can exploit aligners for chewing in order to obtain a better fit of the plastic material to the dental surface and to attachments. This could also help to reduce treatment times. 

Although the article showed that clear aligners do not impair the chewing function, there are still certain things to keep in consideration. It is crucial to assess any potential deformations of the clear aligners caused by the patients’ occlusal stresses during chewing cycles. In addition to the deterioration of the clear aligners, it is crucial to assess the amount of wear on specific components like the attachments. These tools are also vulnerable to biting forces during the chewing cycle, which, over time, may alter their form and, consequently, their functionality. Also, it has been observed that clear aligners can lessen the occurrence of white spots by encouraging improved dental hygiene.

The procedure was validated after performing a statistical comparison between the control groups, which were studied independently at our university and at the University of Bern. This creates new study prospects regarding masticatory function analyses for quantifying the benefits and opportunities mentioned above.

## Figures and Tables

**Table 1:** Comparison of the degree of mixing without aligners based on the number of chewing cycles.

Paired Samples *t*-Test					
			Statistic	df	*p*
H_SD(VOH)5	H_SD(VOH)5	Student’s *t*	6.97	19.0	<0.001
	H_SD(VOH)20	Student’s *t*	15.45	19.0	<0.001
H_SD(VOH)10		Student’s *t*	7.63	19.0	<0.001

**Table 2:** Schemes follow the same formatting.

Paired Samples *t*-Test					
			Statistic	df	*p*
H_SD(VOH)5a	H_SD(VOH)10a	Student’s *t*	3.45	19.0	0.003
	H_SD(VOH)20a	Student’s *t*	12.60	19.0	<0.001
H_SD(VOH)10a		Student’s *t*	7.35	19.0	<0.001

**Table 3:** Comparison of the degree of mixing between the group with and without aligners with five chewing cycles.

Descriptives														
	n_plxel5	H_means5	H_SD(VOH)5	S_mean5	S_SD5	I_mean5	I_SD5	n_pixel5a	H_mean5a	H_SD(VOH)5a	S_mean5a	S_SD5a	I_mean5a	I_SD5a
N	20	20	20	20	20	20	20	20	20	20	20	20	20	20
Missing	0	0	0	0	0	0	0	0	0	0	0	0	0	0
Mean	392,600	0.117	0.552	0.392	0.227	0555	0.0861	379,501	0.106	0.518	0.413	0.226	0.542	0.0824
Median	125,352	0.114	0.571	0.342	0.177	0.554	0.0867	134,677	0.0977	0.548	0.357	0.174	0.544	0.0799
Sandard deviation	519,318	0.0244	0.104	0.115	0.0935	0.0246	0.0135	467,279	0.0219	0.103	0.125	0.0945	0.0237	0.0130
Minimum	61,883	0.0853	0.355	0.281	0.161	0.505	0.0645	93,077	0.0717	0.371	0.292	0.161	0.488	0.0614
Maximum	1,758,691	0.173	0.690	0.617	0.398	0.588	0.110	1,412,444	0.154	0.691	0.704	0.390	0.574	0.109

**Table 4:** Comparison of the degree of mixing between the group with and without aligners with five chewing cycles.

Paired Samples *t*-Test					
			Statistic	df	*p*
H_mean5	H_mean5a	Student’s *t*	1.529	19.0	0.143
H_SD(VOH)5	H_SD(VOH)5a	Student’s *t*	1.047	19.0	0.308
S_mean5	S_mean5a	Student’s *t*	−2.332	19.0	0.031
S_SD 5	S_SD 5a	Student’s *t*	0.659	19.0	0.518
I_mean5	I_mean5a	Student’s *t*	2.444	19.0	0.024
I_SD 5	I_SD 5a	Student’s *t*	1.562	19.0	0.135

**Table 5:** Comparison of the degree of mixing between the group with and without aligners with 10 chewing cycles.

Descriptives														
	n_pixel10	H_mean10	H_SD(VOH)10	S_mean10	S_SD10	I_ mean10	I_SD10	n_pixel10a	H_man10a	H_SD (VOH)10a	S_mean10a	S_SD10a	I_ mean10a	I_SD10a
N	20	20	20		20	20	20	20	20	20	20	20	29	20
Mwing	0	0	0		0	0	0	0	0	0	0	0	0	0
Mean	436,973	0.0706	0.326	0.449	0.200	0.524	0.0702	418,706	0.0893	0.416	0.443	0.216	0.524	0.0785
Median	140,084	0.0799	0.309	0.381	0.166	0.523	0.0669	138,868	0.0888	0392	0.378	0.172	0.524	0.0790
Standard deviation	576,506	0.0177	0.123	0.133	0.0854	0.0275	0.0125	551,271	0.0189	0.127	0.134	0.0932	0.0269	0.0958
Minimum	78,756	0.0452	0.139	0.316	0.112	0.467	0.0504	78,343	0.0629	0.187	0.327	0.135	0.482	0.0619
Maximum	1,834,910	0.113	0.565	0.731	0.375	0.574	0.101	1,700,265	0.121	0.656	0.703	0.393	0.571	0.102

**Table 6:** Comparison of the degree of mixing between the group with and without aligners with 10 chewing cycles.

Paired Samples *t*-Test					
			Statistic	df	*p*
H_mean10	H_mean10a	Student’s *t*	−2.437	19.0	0.025
H_SD(VOH)10	H_SD(VOH)10a	Student’s *t*	−2.899	19.0	0.009
S_mean10	S_mean10a	Student’s *t*	0.602	19.0	0.554
S_SD10	S_SD10a	Student’s *t*	−2.839	19.0	0.010
I_mean10	I_mean10a	Student’s *t*	−0.103	19.0	0.919
I_SD10	I_SD10a	Student’s *t*	−3.123	19.0	0.006

**Table 7:** Comparison of the degree of mixing between the group with and without aligners with 20 chewing cycles.

Descriptives														
	n_pixel20	H_mean20	H_SD(VOH)20	S_mean20	S_SD20	I_ mean20	I_SD20	n_pixel20a	H_man20a	H_SD (VOH)20a	S_mean20a	S_SD20a	I_ mean20a	I_SD20a
N	20	20	20	20	20	20	20	20	20	20	20	20	20	20
Mwing	D	0	0	0	0	0	0	0	0	D	0	0	0	0
Mean	428,502	0.0573	0.118	0.524	0.150	0.500	0.0554	412,784	0.0664	0.218	0.496	0.179	0.492	0.0652
Median	133,255	0.0582	0.0919	0.443	0.124	0.511	0.0538	127,722	0.0650	0.203	0.431	0.143	0.496	0.0632
Standard deviation	550,353	0.0106	0.0848	0.150	0.0777	0.0267	0.00999	531,570	0.0120	0.0856	0.150	0.0836	0.0250	0.00956
Minimum	97,168	0.0349	0.0312	0.412	0.0751	0.441	0.0427	83,920	0.0472	0.0581	0.334	0.0856	0.444	0.0543
Maximum	1,711,024	0.0772	0.388	0.879	0.346	0.527	0.0833	1,575,381	0.0900	0.397	0.799	0.331	0.529	0.0862

**Table 8:** Comparison of the degree of mixing between the group with and without aligners with 20 chewing cycles.

Paired Samples *t*-Test					
			Statistic	df	*p*
H_mean20	H_mean20a	Student’s *t*	−4.10	19.0	<0.001
H_SD(VOH)20	H_SD(VOH)20a	Student’s *t*	−3.65	19.0	0.002
S_mean20	S_mean20a	Student’s *t*	2.33	19.0	0.031
S_SD20	S_SD20a	Student’s *t*	−1.87	19.0	0.078
I_mean20	I_mean20a	Student’s *t*	1.95	19.0	0.066
I_SD20	I_SD20a	Student’s *t*	−3.51	19.0	0.002

**Table 9:** Descriptive statistics for Groups 1, 2 and 3.

Descriptive			
	Group	H_SD(VOH)	Age
N	1	20	20
	2	20	20
	3	20	20
Mean	1	0.107	24.8
	2	0.118	25.4
	3	0.218	25.4
Median	1	0.0901	25.0
	2	0.0919	26.0
	3	0.203	26.0
Standard Deviation	1	0.0852	1.62
	2	0.0848	2.61
	3	0.0856	2.61
Minimum	1	0.0164	22
	2	0.0312	20
	3	0.0581	20
Maximum	1	0.274	29
	2	0.388	30
	3	0.397	30
Shapiro–Wilk W	1	0.878	0.927
	2	0.838	0.967
	3	0.979	0.967
Shapiro–Wilk p	1	0.016	0.136
	2	0.003	0.686
	3	0.924	0.686

**Table 10:** One-way ANOVA between Groups 1, 2 and 3.

ANOVA One Way (Welch)				
	F	gdl1	hdl2	*p*
H_SD(VOH)	10.005	2	38.0	<0.001
Age	0.800	2	35.8	0.457
Sex	0.000	2	38.0	1.000

**Table 11:** Test of homogeneity of variances between Groups 1, 2 and 3.

Test of Homogeneity of Variances (Levene)				
	F	gdl1	hdl2	*p*
H_SD(VOH)	0.249	2	57	0.781
Age	2.698	2	57	0.076
Sex	1.98 × 10^−29^	2	57	1.000

**Table 12:** *t*-test between Groups 1 and 2.

		Statistiche	gdl	*p*
H_SD(VOH)	*t* di Student	−0.418	38.0	0.678
Age	*t* di Student	−1.021	38.0	0.314
Sex	*t* di Student	0.000	38.0	1.000

## Data Availability

The original contributions presented in the study are included in the article, further inquiries can be directed to the corresponding authors.

## References

[1] Buser R., Ziltener V., Samietz S., Fontolliet M., Nef T., Schimmel M. (2018). Validation of a purpose-built chewing gum and smartphone application to evaluate chewing efficiency. J. Oral Rehabil..

[2] van der Bilt A., Engelen L., Pereira L.J., van der Glas H.W., Abbink J.H. (2006). Oral physiology and mastication. Physiol. Behav..

[3] Visscher C.M., Lobbezoo F., Naeije M. (2004). Comparison of algometry and palpation in the recognition of temporomandibular disorder pain complaints. J. Orofac. Pain..

[4] Steele C.M., Van Lieshout P. (2009). Tongue movements during water swallowing in healthy young and older adults. J. Speech Lang. Hear. Res..

[5] Kiliaridis S., Kjellberg H., Wenneberg B., Engstrom C. (1993). The relationship between maximal bite force, bite force endurance, and facial morphology during growth: A cross-sectional study. Acta Odontol. Scand..

[6] Abbate G.M., Caria M.P., Montanari P., Mannu C., Orru G., Caprioglio A., Levrini L. (2015). Periodontal health in teenagers treated with removable aligners and fixed orthodontic appliances. J. Orofac. Orthop..

[7] Levrini L., Bocchieri S., Mauceri F., Saran S., Carganico A., Zecca P.A., Segu M. (2023). Chewing Efficiency Test in Subjects with Clear Aligners. Dent. J..

[8] Zachrisson B.U., Zachrisson S. (1971). Caries incidence and orthodontic treatment with fixed appliances. Scand. J. Dent. Res..

[9] Chapman J.A., Roberts W.E., Eckert G.J., Kula K.S., Gonzalez-Cabezas C. (2010). Risk factors for incidence and severity of white spot lesions during treatment with fixed orthodontic appliances. Am. J. Orthod. Dentofac. Orthop..

[10] Moshiri M., Eckhart J.E., McShane P., German D.S. (2013). Consequences of poor oral hygiene during aligner therapy. J. Clin. Orthod..

[11] Levrini L., Mangano A., Margherini S., Tenconi C., Vigetti D., Muollo R., Marco Abbate G. (2016). ATP Bioluminometers Analysis on the Surfaces of Removable Orthodontic Aligners after the Use of Different Cleaning Methods. Int. J. Dent..

[12] Yan D., Liu Y., Che X., Mi S., Jiao Y., Guo L., Li S. (2021). Changes in the Microbiome of the Inner Surface of Clear Aligners after Different Usage Periods. Curr. Microbiol..

[13] de Abreu R.A., Pereira M.D., Furtado F., Prado G.P., Mestriner W., Ferreira L.M. (2014). Masticatory efficiency and bite force in individuals with normal occlusion. Arch. Oral Biol..

[14] Shim J., Ho K.C.J., Shim B.C., Metaxas A., Somogyi-Ganss E., Di Sipio R., Cioffi I. (2019). Impact of post-orthodontic dental occlusion on masticatory performance and chewing efficiency. Eur. J. Orthod..

[15] Lucchese A., Gherlone E. (2013). Prevalence of white-spot lesions before and during orthodontic treatment with fixed appliances. Eur. J. Orthod..

[16] Sheridan J.J., Armbruster P., Moskowitz E., Nguyen P. (2001). Avoiding demineralization and bite alteration from full-coverage plastic appliances. J. Clin. Orthod..

[17] Lazar L., Vlasa A., Beresescu L., Bud A., Lazar A.P., Matei L., Bud E. (2023). White Spot Lesions (WSLs)—Post-Orthodontic Occurrence, Management and Treatment Alternatives: A Narrative Review. J. Clin. Med..

[18] Lyros I., Pavi E., Tsolakis A., Makou M., Kyriopoulos J. (2019). Satisfaction with Orthodontic Care Provided in a University Orthodontic Clinic. Open Dent. J..

[19] Ikebe K., Matsuda K., Morii K., Furuya-Yoshinaka M., Nokubi T., Renner R.P. (2006). Association of masticatory performance with age, posterior occlusal contacts, occlusal force, and salivary flow in older adults. Int. J. Prosthodont..

[20] Schimmel M., Voegeli G., Duvernay E., Leemann B., Muller F. (2017). Oral tactile sensitivity and masticatory performance are impaired in stroke patients. J. Oral Rehabil..

[21] Weijenberg R.A., Lobbezoo F., Visscher C.M., Scherder E.J. (2015). Oral mixing ability and cognition in elderly persons with dementia: A cross-sectional study. J. Oral Rehabil..

[22] Endo T., Komatsuzaki A., Kurokawa H., Tanaka S., Kobayashi Y., Kojima K. (2014). A two-colored chewing gum test for assessing masticatory performance: A preliminary study. Odontology.

[23] Liedberg B., Owall B. (1995). Oral bolus kneading and shaping measured with chewing gum. Dysphagia.

[24] Prinz J.F. (1999). Quantitative evaluation of the effect of bolus size and number of chewing strokes on the intra-oral mixing of a two-colour chewing gum. J. Oral Rehabil..

[25] Schimmel M., Christou P., Herrmann F., Muller F. (2007). A two-colour chewing gum test for masticatory efficiency: Development of different assessment methods. J. Oral Rehabil..

[26] Schimmel M., Christou P., Miyazaki H., Halazonetis D., Herrmann F.R., Muller F. (2015). A novel colourimetric technique to assess chewing function using two-coloured specimens: Validation and application. J. Dent..

[27] Speksnijder C.M., Abbink J.H., van der Glas H.W., Janssen N.G., van der Bilt A. (2009). Mixing ability test compared with a comminution test in persons with normal and compromised masticatory performance. Eur. J. Oral Sci..

